# Genotoxic Potential of Anthropized Water Bodies in the Hanoi Region of Vietnam Assessed with the Comet Assay on Erythrocytes of Nile Tilapia (*Oreochromis niloticus*)

**DOI:** 10.1007/s00128-025-04023-y

**Published:** 2025-03-13

**Authors:** Elodie Pepey, Gwenn Pulliat, Truong Dinh Hoai, Michaël Bruckert, Geneviève Conéjéro, David Boggio, Coline Perrin, Mathilde Valette, Simon Pouil

**Affiliations:** 1 ISEM, Univ Montpellier, CNRS, IRD, CIRAD, Montpellier, France; 2 CIRAD, UMR AGAP Institut, Montpellier, F-34398 France; 3 UMR AGAP Institut, Univ Montpellier, CNRS, INRAE, Institut Agro, Montpellier, France; 4 CNRS, UMR ART-Dev, University of Montpellier, CNRS, Université Paul Valéry Montpellier 3, Université de Perpignan Via Domitia, CIRAD, Montpellier, France; 5 Faculty of Fisheries, Vietnam National University of Agriculture, Hanoi, Vietnam; 6 CIRAD, UMR INNOVATION, Hanoi, Vietnam; 7 INNOVATION, Univ Montpellier, CIRAD, INRAE, Institut Agro Montpellier, Montpellier, France; 8 IPSiM, Univ Montpellier, CNRS, Institut Agro Montpellier, INRAE, Montpellier, France; 9 Department of Information Technologies, CIRAD, Montpellier, France; 10 CIRAD, US 49 Analyses, Montpellier, France; 11 Univ Montpellier, CIRAD, Analyses, Montpellier, France; 12 Université Paris-Saclay, INRAE, AgroParisTech, Jouy-en-Josas, GABI France

**Keywords:** DNA damage, Fish, Freshwater, Genotoxicity, Trace elements, Vietnam

## Abstract

The Black and Nhue-Day River sub-basins near Hanoi, Vietnam, are crucial aquatic ecosystems that are suffering from severe pollution stemming from industrial, domestic, and agricultural sources, which pose risks to environmental and public health. We assessed water genotoxicity at four locations along a gradient of urbanization in Hanoi and its peripheral regions: a fish farm at Hoa Binh reservoir (HB), a peri-urban fish farm in Phu Xuyen district (PX), and urban lakes Truc Bach (TB) and Thien Quang (TQ). Using the comet assay on Nile tilapia erythrocytes, DNA damage (% tail DNA), reflecting fragmented DNA that migrates out of the nucleus during electrophoresis, demonstrated significant differences between sites (*p* < 0.001). Urban lakes exhibited lower damage (TB: 16 ± 10%, TQ: 33 ± 17%), while the highest damage levels were observed in the hydropower reservoir (HB: 70 ± 15%). Trace elements (i.e., As, Cd, Cr, Ni, and Pb) analyzed in water did not exhibit a significant correlation with DNA damage, suggesting the presence of other unexamined contaminants, such as pesticides, that may explain these findings. These genotoxicity results emphasize the need for further research to identify the specific origins of the observed DNA damage, such as potential contributions from agricultural runoff, untreated wastewater, or other unexamined contaminants. Understanding these sources is essential for developing targeted water management practices to mitigate environmental risks and ensure the safety of aquaculture products, particularly in areas like the HB reservoir, where fish farming supports food security.

## Introduction

Aquatic ecosystems worldwide are increasingly threatened by the influx of untreated domestic wastewater, mining effluents, industrial discharge, metallurgical waste, and agricultural runoff. In Vietnam, despite efforts to enforce environmental safeguards, water pollution continues to escalate, driven by rapid urbanization, intensified agricultural practices, industrial expansion, and population growth (Hoi 2020). For example, studies have highlighted that surface waters in Hanoi frequently exceed national standards for heavy metals such as Pb, and untreated wastewater remains a major contributor to pollution (Nguyen et al. 2022). Water pollution is particularly severe in densely populated suburban areas of major cities, where insufficient regulations and underdeveloped water infrastructure exacerbate the problem (Hoi 2020; Wright-Contreras et al. 2017). Compounding the situation is the rising volume of untreated wastewater from both domestic and industrial sources that are directly released into aquatic environments (Marcussen et al. 2008; Trang et al. 2022) while investment in water management infrastructure lags behind the growing demand for robust water treatment systems (Pham et al. 2023).

The Nhue-Day River sub-basin, which encompasses Hanoi, has been the focus of numerous investigations highlighting severe levels of contamination. Studies have reported high levels of physical, chemical, and biological pollutants across various locations within the basin (Luu et al. 2021; Ngo et al. 2021). Regarding non-essential trace elements, Nguyen et al. (2019), noted frequent exceedances of national standards set by the National Technical Regulation on Surface Water Quality (QCVN 08:2023/BTNMT) for As (0.2–131 µg L^− 1^), Cd (2.1–18 µg L^− 1^) and Hg (0.11–4.1 µg L^− 1^) and highlighted that As was identified as the most important pollutant causing both non-carcinogenic and carcinogenic concerns. Similarly, Ngo et al. (2021) found that the concentrations of non-essential elements (Pb and Cd) surpassed the limit values provided by the National Technical Regulation on Surface Water Quality for Protection of Aquatic Lifes (QCVN 38:2011/BTNMT) at most sampled locations, particularly during the spring. These findings underscore the pressing need to assess the ecological impact of such contamination.

Aquatic pollution exposes organisms to a range of xenobiotics, including genotoxic agents such as non-essential trace elements, which have been shown to induce DNA damage in aquatic species, particularly fish (Lawrence and Hemingway 2008). Unrepaired DNA damage leads to metabolic impairments, growth inhibition, and reduced reproductive success, ultimately affecting population structures and ecosystem stability (Bickham et al. 2000; Pavlica et al. 2011). Therefore, assessing DNA damage is of primary importance for evaluating the impact of pollution on aquatic communities and identifying contaminant sources, such as non-essential trace elements and pesticides, that may accumulate in fish and pose risks to both human health and food security.

The alkaline comet assay, initially developed by Östling and Johansson (1984) and later refined by Singh et al. (1988), is a highly sensitive method for detecting DNA damage. Its ability to detect early-stage genotoxic effects with minimal sample requirements makes it a valuable tool for environmental research (Christofoletti et al. 2009), especially in pollution-related studies in aquatic ecosystems (Jiang et al. 2023). Although less standardized and unable to detect complex chromosomal aberrations compared to the micronucleus assay, the comet assay remains a reliable and adaptable method for environmental biomonitoring, as demonstrated in various studies, including those conducted in low-income settings (e.g., Pepey et al. 2023).

Fish species are commonly employed as model organisms in genotoxicity studies due to their ability to accumulate pollutants and respond to low contaminant concentrations (Frenzilli et al. 2009; Russo et al. 2004). Among these, Nile tilapia (*Oreochromis niloticus*), a primary species cultured and consumed in the Red River Delta (Tri et al. 2022), serves as an excellent bioindicator. The expanding production of tilapia in regions undergoing rapid urbanization, industrialization, and agricultural intensification makes this species particularly relevant for assessing the genotoxic effects of environmental contamination.

The objectives of this study are twofold: first, to evaluate genotoxicity in the Red River basin using the alkaline comet assay on Nile tilapia erythrocytes; and second, to investigate the potential link between genotoxic effects and trace element concentrations (As, Cd, Cr, Ni, and Pb) in water samples collected from four different sites. We hypothesize that diverse anthropogenic activities surrounding the sampling sites, including urban wastewater discharge, agricultural runoff, and pesticide use, contribute to genotoxic effects in exposed organisms along a gradient from urban to rural areas around Hanoi. This research aims to provide critical insights into the ecological and health implications of pollution in thisregion.

## Materials and Methods

Sampling was conducted in October 2019, at the onset of the dry season. This period is characterized by more stable environmental conditions and relatively low fluctuations in surface water contaminant concentrations including As, Cd and Pb (Nguyen and Huynh 2022). Only one set of samples was collected, as access to the various sampling sites was very restrictive and required prior permission from local authorities.

### Sampling Locations

We focused on two sub-basins of the Red River watershed: the Black River and the Nhue-Day River sub-basins. The Black River (Song Da in Vietnamese) is the main tributary of the Red River, joining it about 50 km upstream of Hanoi. Its watershed covers 53,000 km² across China and Vietnam, characterized by sloping agricultural lands and degraded forests. The Nhue-Day sub-basin, covering 7,665 km² (Duong et al. 2016), has a dense network of canals and plays a crucial role in supporting 12 million inhabitants through freshwater aquaculture and agriculture (Ngo et al. 2021). The Black River is prone to agricultural contamination, particularly pesticides (Vu et al. 2023), while the Nhue-Day sub-basin faces well-documented pollution from wastewater and industrial discharge (Ngo et al. 2021).

To explore different pollution types across urban and rural settings, we selected four sampling locations including farmed fish from cages in the Hoa Binh reservoir (Black River) and earthen ponds in Phu Xuyen (Nhue-Day River), and wild-caught tilapia from inner-city lakes. The Hoa Binh reservoir, established in 1989 by damming the Black River, serves multiple purposes, including electricity generation and irrigation (Vinh et al. 2014). Aquaculture has since expanded with cage farms appearing in various parts of the lake. Previous studies have shown that the dam has significantly altered sediment distribution downstream, affecting contaminant transport (Dang et al. 2010; Thanh et al. 2008; Vinh et al. 2014). Sediment often carries trace element contaminants, influencing their deposition (Roberts et al. 2022) which in turn impacts their bioavailability in aquatic ecosystems.

In Phu Xuyen, tilapia were collected from a peri-urban fish farm where fish are reared in earthen ponds. The ponds are filled with water pumped from irrigation canals, which are connected to the nearest river. This water circulates through the fields (rice, vegetables…) before being used in the ponds, thus these farms are closely exposed to pollutants from these fields. Additionally, Phu Xuyen is located downstream of the city, thus exposed to urban wastewater since almost 80% of domestic wastewater is directly discharged into the environment without treatment (Trang et al. 2022). Finally, the southern region of Hanoi province is experiencing a rapid expansion and intensification of its aquaculture production, which currently occupies nearly 15% of farmland in Hanoi. These farmlands, formerly rural, are now part of a peri-urban zone mixing agriculture, industry, and residential areas.

The third location was in Hanoi’s city core, where 120 lakes and ponds play a crucial role in stormwater management and serve as recreational spaces for local communities. Seventeen of these lakes, located in the inner city (Nguyen et al. 2016), are heavily impacted by domestic wastewater, highlighting their dual role as both wastewater receptacles and vital urban infrastructures. Wild tilapia thrive in these organic matter-rich environments, which can promote trace element bioaccumulation in aquatic organisms (Zeng et al. 2024), and residents often release fish fry to support fishing activities. We sampled tilapia from TB Lake, near West Lake, and from TQ Lake, located just south of TB Lake.

### Water Analysis

Composite samples were created by pooling water collected from three sampling points at each study site, taken at the same time as the tilapia used for genotoxicity testing. Sampling points were chosen based on their proximity to likely contamination sources and their representativeness of the overall site conditions based on input from local collaborators. At each location, water samples were collected on-site, filtered using 0.2 μm PES filters, and stored in 50 mL Falcon^®^ tubes with trace element grade nitric acid (pH < 2) for the analysis of trace metals. All samples were kept at room temperature (~ 20 °C) in the dark until analysis.

Concentrations of particulate (from filters) and dissolved (from filtered water) trace elements, including As, Cd, Cr, Ni, and Pb, were measured as potential genotoxic agents (Cambier et al. 2010; Salem et al. 2014). These trace elements were analyzed directly in the water samples stored in the Falcon^®^ tubes using an ICP-MS (ICAP Q, Thermo Scientific). Any trace metals remaining on the filters were digested according to ISO 14869-1 (V 2001) method using a mixture of concentrated nitric, hydrofluoric, and perchloric acids (analysis grade). The accuracy of the analytical procedure was tested and validated by analyzing certified reference materials SLRS6 (River water) and CRM 7004 (soil Metranal^®^ 34). Each batch of samples included three procedural blanks and four samples of certified materials (one solution and three soils), which were treated and analyzed similarly to the samples. The ICP-MS calibration standards were prepared from a certified solution containing several elements at 10 µg L^− 1^ from SCP Sciences. Recoveries from reference materials were in the range of 90–110% for SLRS6 (except for Cd due to the low Cd concentration in the reference material used) and 80–120% for CRM 7004. The detection limits for As, Cd, Cr, Ni, and Pb in water were 0.002, 0.001, 0.002, 0.006, and 0.007 µg L^− 1^, respectively. For suspended matter, detection limits ranged as follows: 0.91, 0.24, 1.33, 7.58, and 6.30 mg kg^− 1^ for As, Cd, Cr, Ni, and Pb, respectively.

### Fish Blood Sampling

Fish were captured at each site using a net (*n* = 11–12 per site). The weights of the fish were recorded as follows: 201.3 ± 40.8 g (TQ), 203.2 ± 41.6 g (TB), 265.5 ± 34.0 g (PX), and 305.6 ± 73.8 g (HB). An additional batch of larger fish (i.e., 910.5 ± 173.0 g, *n* = 11 fish), was collected from the peri-urban fish farm (PX) to assess the effects of weight on DNA damage. Fish were transported in the same water and under air bubbling to the Faculty of Fisheries, Vietnam National University of Agriculture (Hanoi, Vietnam) where blood samples were taken the same day. Fish were anesthetized using eugenol at a concentration of 0.5 mL L^− 1^, and blood samples were collected from the ventral tail vein using a syringe containing heparin. No mortality events were observed.

### Comet Assay

The alkaline comet assay was conducted on Nile tilapia erythrocytes, among the most accessible nucleated cells in fish through non-lethal sampling. We followed the protocol specified by Singh et al. (1988) and Osman et al. (2012), with some modifications (Pepey et al. 2023). Frosted microscope slides were pre-coated with 1% (w/v) normal-melt agarose and left to dry at room temperature for at least two days. On the day of sampling, immediately after each blood puncture from the fish, a dilution with phosphate-buffered saline was performed at a ratio of 1:500 (v/v). For each sample, 40 µL of diluted blood sample was mixed with 360 µL of 0.5% (w/v) low-melting agarose and 150 µL of the mixture was placed on the pre-coated slide and covered with a coverslip. The slides were kept at 4 °C for 15 min to facilitate solidification, followed by immersion in cold lysing buffer (2.5 M NaCl, 100 mM ethylenediaminetetraacetic acid [EDTA], 10 mM trishydroxymethylaminomethane [Tris] base, pH 10, 1% Triton X-100, and 10% dimethyl sulfoxide) overnight at 4 °C. The slides were then transferred into electrophoresis buffer (300 mM NaOH, 1 mM EDTA, pH ≥ 13) at 4 °C for 40 min, followed by electrophoresis at 0.8 V/cm and 300 mA at 4 °C for 40 min. A neutralization step was conducted by incubating the samples in 0.4 M Tris buffer (pH 7.50) for three consecutive periods of 10 min each. Slides were rinsed for 5 min in ultra-pure water followed by immersion in ethanol (≥ 99.6%) for 10 min to initiate dehydration before being dried at 45 °C for 1 h. The slides were stained with 100 µL of SYBR^®^ Gold (Thermo Fisher Scientific) stain which was diluted 10,000 times with Tris/EDTA buffer (pH 8.0). The comets were observed using an epifluorescence microscope (Nikon Eclipse NI-E, objective Plan APO 0,75 NA 20x). The OpenComet plugin integrated with ImageJ software (Gyori et al. 2014) was employed for the analysis of two slides, each containing 200 cells (totaling 400 cells per fish) to quantify the % tail DNA (Kumaravel et al. 2009). DNA damage levels were classified based on the criteria established by Mitchelmore et al. (1998) for mussels and adopted by de Souza Trigueiro et al. (2021) for fish: minimal (0–10%), low (10–25%), moderate (> 25–50%), high (> 50–75%), and extreme damage (> 75%). To ensure reproducibility and consistency in comet scoring, all images were initially analyzed by a single observer and then independently reviewed by a second observer to validate their accuracy and reliability.

### Data Analysis

DNA damage between sites was analyzed using ANOVA, followed by Tukey’s test for pairwise comparisons. Fish weight was initially tested as a fixed effect but was excluded from the model due to its lack of significant impact on DNA damage. Assumptions of normality and homoscedasticity were assessed through visual inspection of residual-fit plots. For fish sampled at the peri-urban fish farm (PX), the effect of body weight was evaluated using a Student’s t-test, comparing two weight classes: [200–350[ g and [700–1150[ g. Normality and homoscedasticity were formally tested using the Shapiro-Wilk and Levene’s tests, respectively. Sample sizes were determined to ensure a statistical power of at least 0.8. Post-hoc analyses confirmed the actual power exceeded this target, based on the observed means and standard deviations for each group, using the pwr package.

Additionally, we assessed the potential relationship between DNA damage in fish erythrocytes and contaminant concentrations in the water at each site using Pearson correlations for each analyzed trace element.

All statistical analyses were conducted in R v.4.2.2 (R Development Core Team 2022), with a significance level of α = 0.05. Results are reported as mean ± SD.

## Results and Discussion

### DNA Damages in Fish Across Sampled Sites

**Fig. 1:**
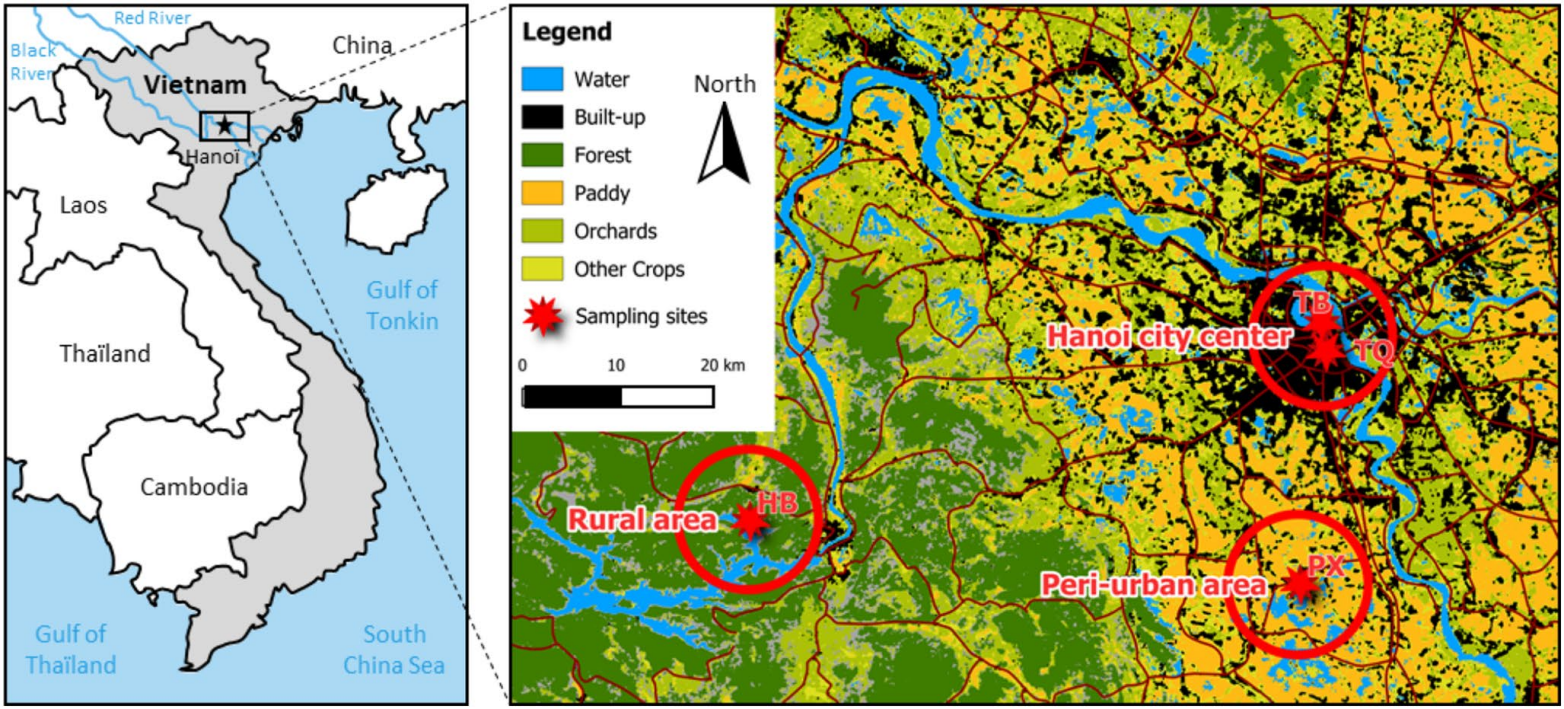
Map of sampling locations: Hoa Binh reservoir (HB) is in a rural area; the fish farm (PX) is located in a peri-urban area; Truc Bach lake (TB) and Thien Quang lake (TQ) are located in Hanoi city center

In this study, we evaluated % tail DNA in Nile tilapia erythrocytes collected from four aquatic sites along a gradient of urbanization in Hanoi and its peripheral regions. The lowest % tail DNA values were recorded in fish from urban lakes (TB: 16.1 ± 10.5%, TQ: 33.0 ± 17.0%), while the highest values were observed in farmed fish from the hydropower reservoir (HB: 70.3 ± 14.7%) (Fig. 1). Intermediate values were detected in fish from the peri-urban fish farm (PX: 44.0 ± 13.1%) (Fig. 1). ANOVA confirmed this gradient (F(3,41) = 28.72, *p* < 0.001), and pairwise comparisons revealed significant differences between DNA damage in fish from the hydropower reservoir (HB) and urban lakes (*p* < 0.001). However, DNA damage in fish from the peri-urban fish farm (PX) was not significantly different from fish collected from the lake TQ (*p* = 0.261).

The alkaline comet assay is widely recognized as an initial indicator of nonspecific DNA damage, serving as a robust biomarker for aquatic environmental monitoring (Frenzilli et al. 2009). Parameters such as tail length, tail moment, and % tail DNA are commonly used to assess DNA strand breakage. Among these, % tail DNA is considered as the most appropriate metric for quantifying DNA strand breakage in fish (Osman et al. 2008). Our findings align with these previous studies, further supporting the utility of % tail DNA as a reliable measure of DNA damage across varied environmental conditions.

### Effects of Age/Size on DNA Damage

**Fig. 2:**
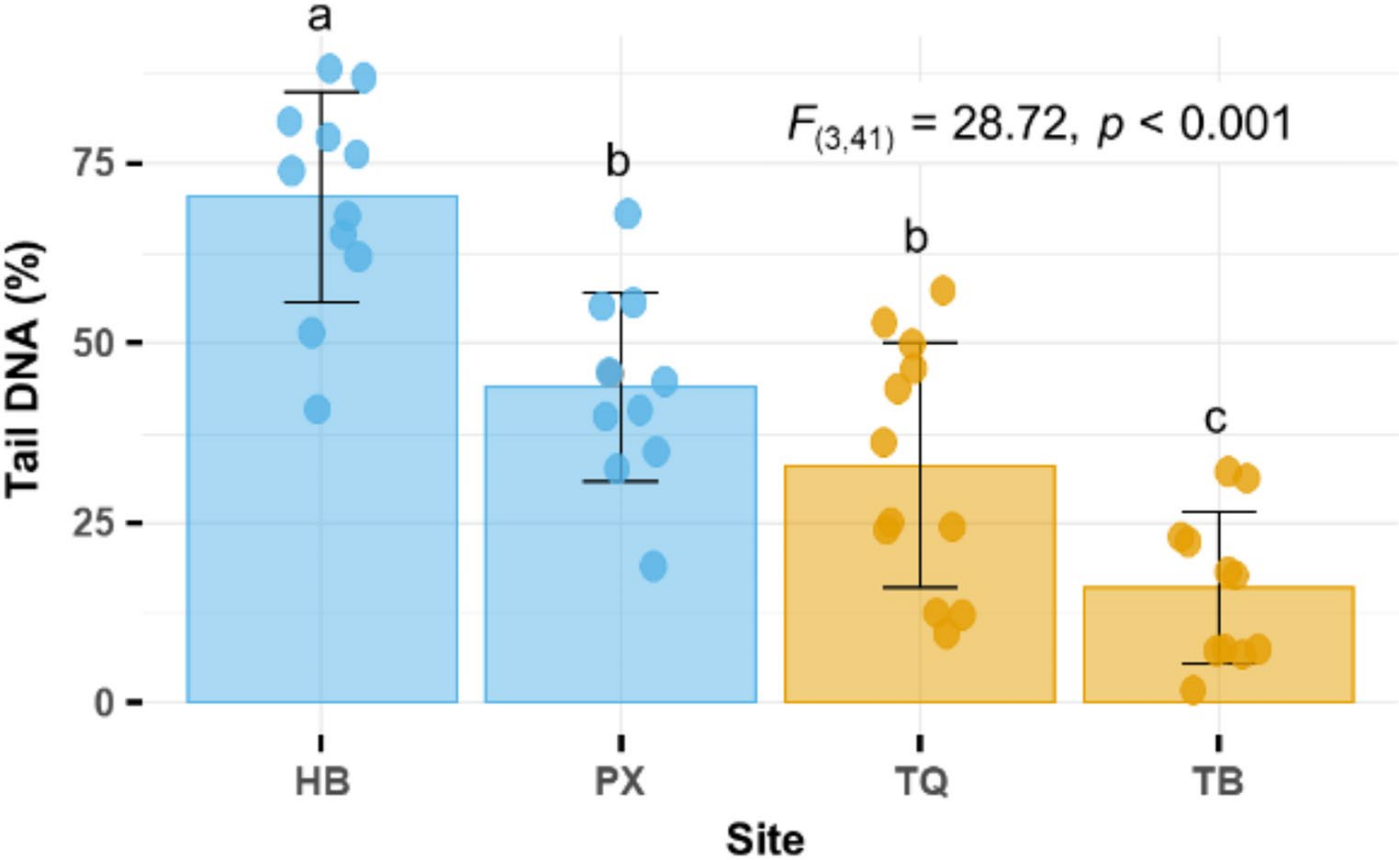
DNA damage (%) to the erythrocytes of farmed Nile tilapia *O. niloticus* (400 measurements per fish) coming from two batches of fish differing in body weight: “small size” batch (mean ± SD: 266 ± 34 g, min-max: 216–328 g, *n* = 11) and “large size” batch (mean ± SD: 953 ± 99 g, min-max: 746–1126 g, *n* = 11)

We did not observe a significant correlation between fish weight and DNA damage across all collected samples. To further investigate this potential relationship, we analyzed the impact of body weight on DNA damage in erythrocytes from two batches of fish collected from the peri-urban fish farm (PX). In the “small size” batch, fish weighed 266 ± 34 g (min-max: 216–328 g), while fish in the “large size” batch weighed 953 ± 99 g (min-max: 746–1126 g). Although larger fish tended to exhibit more severe DNA damage than smaller ones (52.6 ± 22.1% vs. 44.0 ± 13.1%), this difference was not statistically significant (t(16.23) = 1.13, *p* = 0.139) (Fig. 2). These findings suggest that results from the comet assay are consistent across the size range we considered.

Age and size may influence DNA damage due to prolonged exposure to environmental stressors, bioaccumulation of contaminants, and declining cellular repair mechanisms. For instance, oxidative stress, a common driver of DNA damage, increases with age as a result of metabolic changes and the accumulation of reactive oxygen species (ROS) over time (Birnie-Gauvin et al. 2017). Older and larger fish may accumulate more genotoxic contaminants, such as heavy metals or organic pollutants, due to their longer lifespan and greater biomass. Al-Sabti and Metcalfe (1995) suggested that older fish are more sensitive to genotoxic damage due to these cumulative effects. Raphael et al. (2016) observed a significant relationship between fish length and DNA damage in the three-spined stickleback (*Gasterosteus aculeatus*), a Gasterosteidae species, which they attributed to a combination of contaminant bioaccumulation and age-related physiological changes. These findings highlight the importance of considering both age and size when interpreting genotoxicity data in environmental monitoring studies. In contrast, our results indicate that, within the weight ranges analyzed, body size does not significantly influence DNA damage in Nile tilapia. This discrepancy could be attributed to species-specific differences in physiology or detoxification mechanisms, or to the influence of other environmental factors not accounted for in this study.

### Classification of DNA Damages

**Fig. 3:**
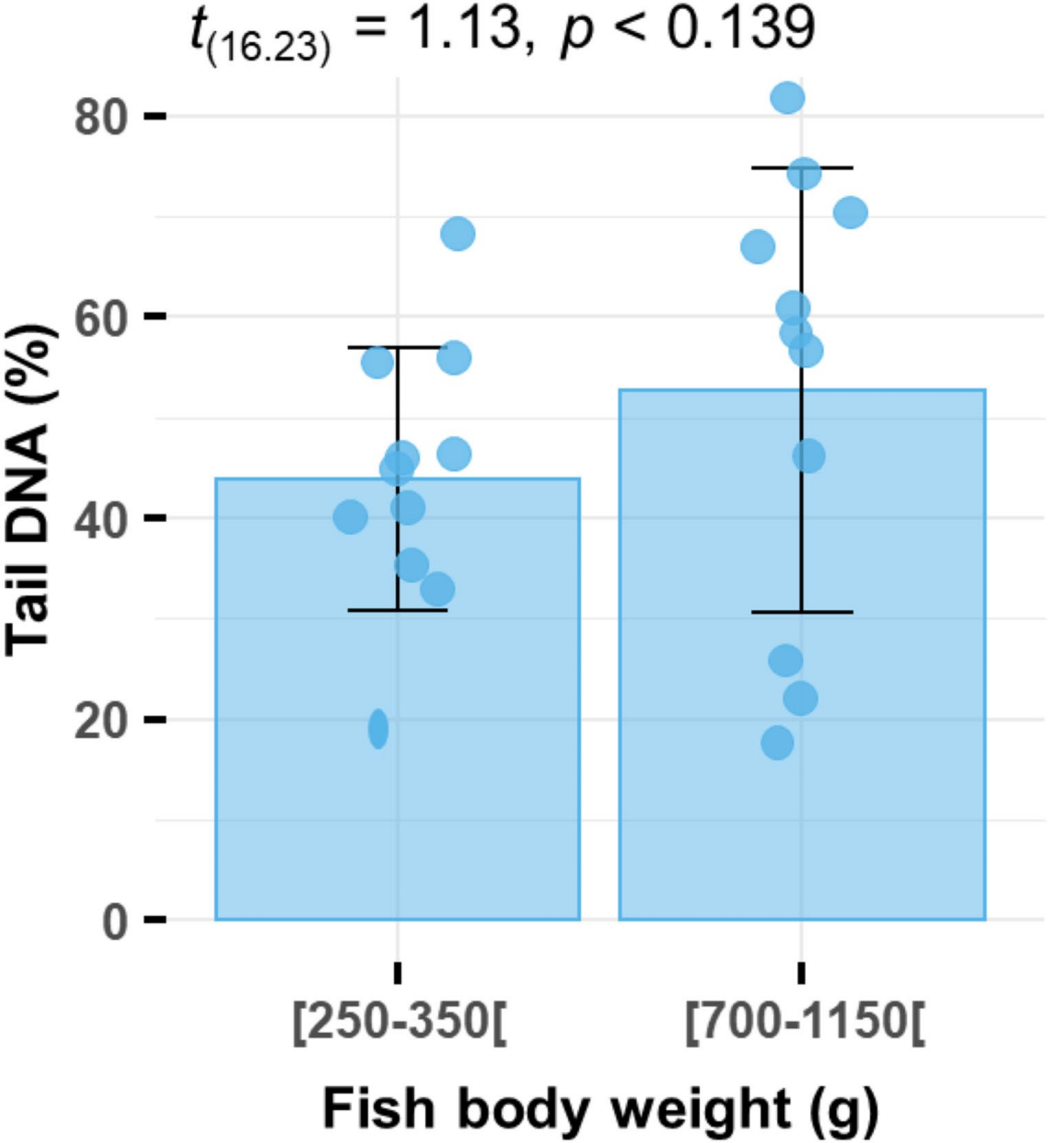
DNA damage (%) to the erythrocytes of farmed Nile tilapia O. niloticus (400 measurements per fish) coming from two batches of fish differing in body weight: “small size” batch (mean ± SD: 266 ± 34 g, min-max: 216-328 g, n = 11) and “large size” batch (mean ± SD: 953 ± 99 g, min-max: 746-1126 g, n = 11)

As a complementary approach to better characterize the level of DNA damage, Mitchelmore et al. (1998) proposed a classification of DNA damage, used later in other studies on fish (Osman et al. 2012). Taking these criteria into account, we determined the distribution of cells according to the category of DNA damage (minimal, low, medium, high, and extreme) at each site (Fig. 3). While 66% of the cells analyzed showed minimal DNA damage at site TB, the opposite was observed at site HB, where 52% and 29% of the cells exhibited extreme and high DNA damage, respectively. The distribution of cells among all damage categories appeared uniform at site PX, with approximately 20% of cells falling into each damage category. This uniformity was also observed at site TQ to a slightly lesser extent. We thus confirmed the extreme damage detected in farmed tilapia collected at the HB site, while conversely, almost no significant damage was found in fish caught at the TB site.

### Possible Sources of Genotoxicity

Taken together, our findings reveal a discernible trend of escalating genotoxicity across distinct urban (TB and TQ), peri-urban (PX), and rural (HB) sites. DNA damage is influenced by many external factors, with exposure to anthropogenically-released chemicals in particular having a significant impact (Lawrence and Hemingway 2008). Several studies have already demonstrated the presence of genotoxicity gradients in various aquatic environments, highlighting a link with observed pollution levels. For instance, Osman et al. (2012) reported a substantial increase in DNA damage observed in the erythrocytes of Nile tilapia and African catfish (*Clarias gariepinus*), a Clariidae species, from heavily polluted areas in the Nile basin. This increase was associated with increased concentrations of several important physicochemical parameters detected in downstream Nile waters, including chemical oxygen demand, phenol, Cd, Cr, Hg and Pb. Similarly, El-Sappah et al. (2022) documented an association between elevated micronuclei frequency and high concentrations of Cd and Pb in various body compartments (gills, liver, and muscles) of Nile tilapia. In Ecuadorian Amazonia, Vasco-Viteri et al. (2023) observed significantly higher numbers of micronuclei and nuclear abnormalities in the blood of Nile tilapia reared in mining areas contaminated with non-essential elements, compared to those raised in non-mining areas. Altogether, these results show that DNA damage is associated, in the wild, with pollution levels when a gradient of pollution exists along the water course. Here, we assume that the progressive increase in the extent of genotoxicity may be associated with increasing pollution along a gradient from urban to rural areas around Hanoi.

We expanded our investigation beyond genotoxicity biomarkers in fish by sampling water to quantify trace element concentrations. Previous studies have highlighted elevated levels of non-essential elements in the Nhue-Day River system’s water and sediments (e.g. Nguyen et al. 2019; Ngo et al. 2021). Among our sampled sites, Hong et al. (2020) detected high As levels (27.74 µg L^− 1^) in water from the TB site, attributed to anthropogenic sources. In the present study, dissolved trace element concentrations (i.e. As, Cd, Cr, Ni, Pb) ranged from below the limits of detection to a maximum of 16 µg L^− 1^ (Table 1) with the hierarchy Cd ⩽ Cr ⩽ Pb ⩽ Ni ⩽ As. Site PX exhibited the highest concentrations of most trace elements, except for As, which peaked at TB. Particulate trace elements were generally below the detection limits, apart from Cr, with concentrations ranging from 6 to 35 mg kg^− 1^. Overall, our trace element measurements did not reveal high concentrations, with the majority of samples below Vietnamese limits or WHO recommendations for drinking water (WHO 2022).

**Table 1:** Concentrations of dissolved (µg L^− 1^) and particulate (mg kg^− 1^) trace elements (i.e., as, cd, Cr, Ni, and Pb) measured in the water (*n* = 1) at the four sampling locations: Hoa Binh reservoir (HB), peri-urban fish farm (PX), Thien Quang lake (TQ) and Truc Bach lake (TB)

Trace element	Dissolved (µg L^− 1^)					Particulate (mg kg^− 1^)			
	HB	PX	TQ	TB		HB	PX	TQ	TB
As	0.94	7.01	4.26	15.83		< LoD	2.20	< LoD	1.00
Cd	0.00	0.05	0.01	0.02		< LoD	< LoD	< LoD	< LoD
Cr	0.17	1.71	0.12	0.31		18.69	27.28	35.36	6.44
Ni	0.12	3.09	3.43	1.32		14.91	14.22	10.90	< LoD
Pb	0.04	3.01	0.27	1.05		< LoD	7.07	< LoD	< LoD

LoD: Limits of detections (see Sect. 2.2 for details)

We did not observe any relationship between DNA damage in fish and the concentrations of dissolved and particulate trace elements in the water. This aligns with existing literature, which suggests that genotoxicity induced by trace elements manifests only at elevated concentrations. For instance, Matsumoto et al. (2006) reported genotoxicity in Nile tilapia exposed for 72 h to Cr-containing tannery effluent, where Cr concentrations reached up to 0.38 mg L^− 1^. More recent findings have emphasized genotoxicity after 72 h of exposure to tannery effluent at 0.1 mg Cr L^− 1^ (Weldetinsae et al. 2017), a concentration still higher than those measured in our study. Several studies have also identified genotoxic effects for As (Ahmed et al. 2011), Cd (Özkan et al. 2011), and Pb (Jiraungkoorskul et al. 2008) in this species but at sublethal concentrations (> 3 mg L^− 1^ for As, > 0.5 mg L^− 1^ for Cd, > 47 mg L^− 1^ for Pb). However, environmental factors may influence the lack of correlation observed in our study. The bioavailability and toxicity of trace elements can be significantly affected by variables such as pH, dissolved organic matter, and interactions with other waterborne chemicals (de Paiva Magalhães et al. 2015), potentially limiting their genotoxic impact. Moreover, genotoxicity in natural environments is often driven by the combined effects of multiple pollutants. The presence of unmeasured contaminants, such as pesticides could contribute to the DNA damage observed in fish, particularly at the PX and HB sites, masking the individual effects of trace elements. Some studies have shown that farmed fish generally have lower contaminant concentrations than their wild counterparts, particularly for semi-persistent and emerging pollutants, as observed in *Boops boops* (Henríquez-Hernández et al. 2017). This difference is attributed to control of feeds in aquaculture. In our study, considering this hypothesis, the significant DNA damage observed in aquaculture fish (PX and HB sites) is likely linked to preferential contamination via dissolved pathway.

**Fig. 4:**
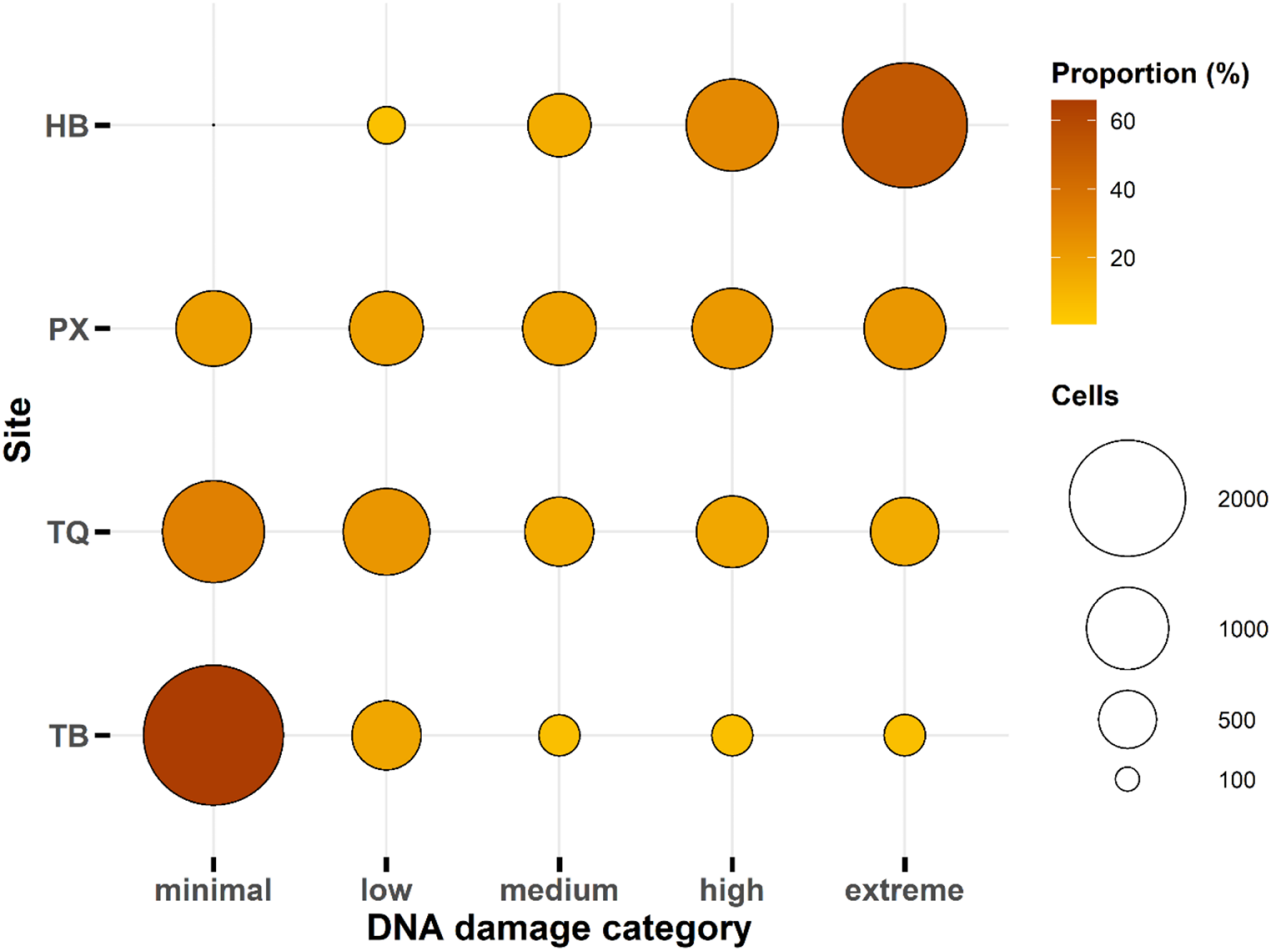
Number of total erythrocyte cells in the different DNA damage categories (i.e. minimal (0–542 10 %), low (>10–25 %), moderate (>25–50 %), high (>50–75 %), and extreme damage (>75 %)) defined 543 according to Mitchelmore et al. (1998) in Nile tilapia O. niloticus collected at the four sampling 544 locations: Hoa Binh reservoir (HB, n = 11), Phu Xuyen fish farm (PX, n = 11), Thien Quang lake (TQ,545 n = 12) and Truc Bach lake (TB, n = 11)

Given this potential role of pesticides, it is important to consider how agricultural land use patterns may influence genotoxicity. In peri-urban and rural areas, where genotoxicity was most pronounced, we propose a link to pesticide exposure, supported by land use patterns. Urban cores like Hanoi likely face minimal agrochemical exposure, contrasting with increased use in less urbanized Red River delta regions. For example, the PX site is surrounded by rice paddies, suggesting high pesticide exposure (Fig. 4). Varied agro-chemical use in peri-urban Hanoi complicates pollutant source identification (Phamova et al. 2022). Establishing a causal relationship between genotoxicity and specific pesticides is challenging (Gendron et al. 2023). Challenges include incomplete pesticide use data, complexities in concentration origins, and interactions within mixtures, making effects hard to predict (ibid.). Hoa Binh reservoir, which receives water from crop cultivation areas, faces potential contamination by pesticides (Salazar and Rand 2020). Hoai et al. (2011) revealed the widespread use of pesticides near Hanoi, including compounds with genotoxic effects such as fenobucarb, trichlorfon, cyfluthrin, and cypermethrin while, recently, Vu et al. (2023) found organophosphorus herbicides in the Red River basin. Pesticide genotoxicity on Nile tilapia is quite well documented. Indeed, Aribisala et al. (2022) noting micronuclei increase in tilapia exposed to paraquat and glyphosate while El-Garawani et al. (2021) and Hathout et al. (2021) highlighted DNA strand breaks after exposure to acetamiprid and imidacloprid while such xenobiotics also impair DNA repair mechanisms (Dey et al. 2023).

## Conclusion

The comet assay on Nile tilapia erythrocytes revealed a gradient of genotoxicity across urban, peri-urban, and rural sites, with the highest % tail DNA observed in fish from the HB hydropower reservoir and PX peri-urban fish farm. These variations suggest differing environmental pressures, likely influenced by agricultural activities near rural sites. While no correlation was found between genotoxicity and trace element concentrations, this may reflect the complex interplay of contaminants or the presence of other genotoxic agents, such as pesticides. Specifically, the results suggest a potential link to commonly used agricultural chemicals like organophosphates or pyrethroids, warranting further investigation. Our findings underscore the need for more comprehensive contamination assessments using advanced ecotoxicity tools, including multi-biomarker approaches and chemical screening techniques. Future studies should explore seasonal and temporal dynamics, additional contaminants such as heavy metals or pharmaceuticals, and their combined effects on aquatic ecosystems. These efforts are crucial for developing evidence-based policies to regulate pesticide use, improve water quality monitoring programs, and promote sustainable agricultural practices in the Hanoi region.
